# Differences between subjective and disability health expectancies across ages in older adults

**DOI:** 10.1038/s41598-024-65416-3

**Published:** 2024-06-26

**Authors:** Antoine Gbessemehlan, Emmanuelle Cambois, Nicolas Brouard, Luc Letenneur, Hélène Amieva, Karine Pérès

**Affiliations:** 1 University of Bordeaux, INSERM, UMR 1219, Bordeaux Population Health Research Center, 146 Rue Léo Saignat, 33076 Bordeaux, France; 2 National Institute of Demographic Studies (INED), Paris, France

**Keywords:** Public health, Epidemiology, Quality of life, Geriatrics

## Abstract

Health expectancies (HEs) have become a key indicator for monitoring healthy aging. So far, they have mainly been calculated based on functional rather than subjective health measures. Yet, by integrating several dimensions (medical, social, and cultural), subjective health is also an important measure of an older person’s health status. In this study, we first estimated HEs using self-rated health (SRH), by age and sex. Second, we compared these results to those obtained when using a disability measure. We used pooled data from three prospective population-based cohorts including adults aged 65 years and over, living in Southwestern France (N = 4468). SRH was assessed using a single question and disability was measured using the Lawton scale. Healthy/Unhealthy Life Expectancies (HLE/UHLE) and Disability/Disability-Free Life Expectancies (DLE/DFLE) were estimated using the Interpolated Markov Chain program (IMaCh), separately in men and women. Women lived longer than men, with similar HLE but longer UHLE at all ages. The proportion of HLE in total LE decreased with age for both sexes and for women, it became smaller than the proportion of UHLE from age 73 onward. In both sexes, while the DLE was shorter than the UHLE in the youngest, a reversal was observed with advancing age. This change occurred earlier in women. Our study supports that SRH and disability showed different aging patterns, with sex and age differences. From a public health perspective, SRH and disability indicators appeared not interchangeable as they uncovered complementary but different information on the needs of aging people.

## Introduction

The increase in Total Life Expectancy (TLE) in most countries is good news and a real opportunity for society to continue to benefit from the significant contributions of older adults through their involvement in formal and informal work, volunteering, roles within the family^1^. However, this situation is also challenging because these extra years of life are not necessarily lived in good health as the three well-known scenarios of aging (expansion of morbidity, compression of morbidity, and the dynamic equilibrium) show^2^. In addition, numerous studies highlighted sex-specificity, both in the number of expected life years and in the quality of health conditions^3,4^. Evidence confirms that women live longer but spend more years in poor health than men on many health dimensions^3,4^. These sex disparities are mainly related to the different risks of suffering from disabling chronic conditions, comorbidities, and lethal diseases, which are also the result of differences in the life course of men and women (e.g. behaviors, health practices, occupational exposures)^5–8^. In this context, Health Expectancies (HEs) indicators have emerged as relevant summary measures of population health and are increasingly used to highlight and monitor sex differences, alongside increases in TLE^3,4,9^. HEs estimate the number of years to be lived with and without a given health condition within TLE^9,10^. As TLE differs across (sub)populations, it is relevant to assess the extent to which the additional life years gained are spent in good or poor health, and how the patterns change with the health measures considered. There are as many HEs as there are health measures, but Disability and Disability-Free Life Expectancy (DLE/DFLE) are the most common^10,11^.

Disability scales assess an individual’s capacity to perform basic or Instrumental Activities of Daily Living (ADL or IADL) due to deteriorating health and the sudden or progressive onset of impairments and functional limitations^12,13^. Studies have shown that whatever the disability indicator considered, women spend more years with disability than men^14–17^, suggesting that women pay for their longer life by poorer health. Disability accumulates with age, while recovery capacity decreases with the intensity of the limitations^18–20^. Designed to meet urgent needs of assistance, these measures address crucial public health concerns in the monitoring of dependency and long-term care. However, alternative health indicators, such as subjective measures (e.g., self-rated health), uncover other meaningful facets of the health of older people^21,22^.

Self-rated health (SRH) appears as the most inclusive and informative measure of an individual’s overall health status. According to the model illustrating the process of individual health assessment, it has been shown that the measure of SRH reflects several dimensions of health^21^. Indeed, when assessing their health, individuals take into account relevant information about their age, their medical condition, the perceived gap between current and past health conditions, the comparison with other people they know in their age group, and general expectations^21^. This assessment is also influenced by cultural characteristics (norms and values) that make individuals more or less likely to express positive feelings. Therefore, this indicator based on the individual’s perception is the result of a complex construction and has been proved to be a predictor of mortality and several adverse health outcomes^23–25^. This predictive effect does not disappear when controlling for objective health factors^23,24^. Although studies report a higher frequency of poor SRH in women than in men^5,24,25^, as with disability, these conditions do not fully overlap and capture complementary facets of aging and of the individual health status. Indeed, with advancing age, the presence/absence of disability is not closely equivalent to poor/good SRH, particularly among very old adults, some of whom remain satisfied with their health despite the onset of a disability or serious health events^26–28^. This paradox, which illustrates such a shift in SRH response with age, reinforces the fact that DFLE and DLE do not allow to fully capture the time spent in good or poor SRH in older adults. To date, research on HEs based on subjective health measures is less common than that based on disability, and there is little evidence on sex differences in subjective Healthy or Unhealthy life expectancy (HLE or UHLE)^3,29,30^. Furthermore, we do not know whether there are any sex patterns in subjective HEs across ages, and whether there are differences when considering subjective or disability indicators (this latter being considered as a more objective one).

To add to the HEs literature, the present study aims to examine sex differences in HLE and UHLE across ages in the French older population using data from three population-based cohorts. In addition, we compared the sex-specific dynamics in HEs calculated using the subjective and the disability indicators across ages.

## Methods

### Data and study population

We used data from three prospective population-based cohorts on aging (PAQUID, Three-City (3C), and AMI) initially including older adults aged 65 years and over and living in southwestern France^31–33^. These cohorts have similar design and methodology (specificities are detailed below). Data were collected using standardized questionnaires during face-to-face interviews at the participants’ home by trained neuropsychologists. The follow-up visits were performed every 2–3 years using the same data collection procedures and vital status with the exact date of death was regularly updated over time. All participants gave written informed consent before inclusion and the three studies have been approved by an ethic committee (of University Hospital of Bordeaux for PAQUID and for AMI; and of the Kremlin-Bicêtre University Hospital and Sud-Mediterranée III committee for 3C). These research and all methods were performed in accordance with the relevant guidelines and regulations embodied in the Declaration of Helsinki.

The PAQUID (‘‘Personnes Agées QUID’’) cohort aimed to study cognitive and functional aging^31^. It started in 1989 and initially included 3777 people randomly selected from the electoral rolls (this sampling frame excludes the 5% of French voters who are not registered and the foreign population). Participants have been followed for more than 30 years. In the current study, to analyze a same period (i.e., from the 2000’s), we decided not to consider the oldest data of the cohort, i.e., those collected over the 1990’s. As a result, our baseline time was the follow-up visit conducted in 1999–2000 (i.e., 10 years after PAQUID started). We therefore excluded participants who died/drop out (n = 2329) before this visit. Finally, after excluding the 59 participants who did not have information on the SRH/disability variables at any wave of data collection, the PAQUID sample included 1,389 participants aged 75 and over and followed over 20 years (i.e., nine data collection waves).

The 3C cohort started in 1999, had set-up in three French cities (Bordeaux, Dijon, and Montpellier) and aimed to investigate vascular risk factors of dementia^32^. Participants were also selected from the electoral rolls and followed-up over 17 years (nine data collection waves). Because of discontinued follow-ups in the Montpellier and Dijon centers, the present study only included the participants living in Bordeaux (n = 2104). Of them, 2101 were considered after exclusion of three participants who had missing data on SRH/disability variables for all collection waves.

**Figure 1:**
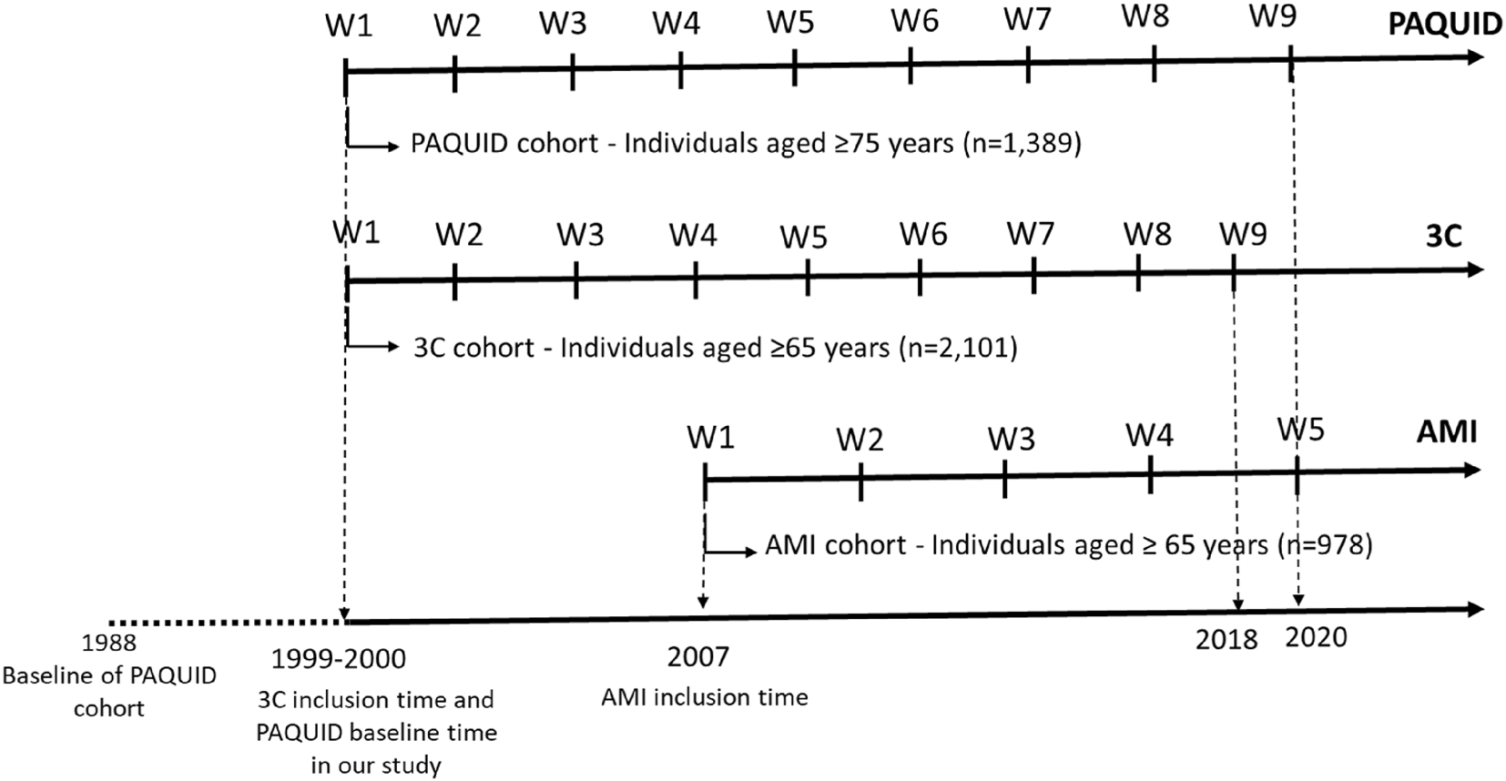
Timeline of data collection waves in each cohort.

The AMI (Aging Multidisciplinary Investigation) cohort started in 2007 and aimed to study health and aging in retired farmers living in rural area^33^. Participants were randomly recruited from the reimbursement database of the French Farmer Health Insurance System, the unique and exhaustive national database covering all farmers insured in their own name. Participants enrolled in AMI cohort were followed-up over 10 years (five data collection waves). Among the 1002 participants, 978 were analyzed after exclusion of 24 subjects for missing data (on date of death, SRH/disability variables for all data collection waves). The Fig. 1 presents the timeline for the waves of data collection in each cohort.

### Measures

#### Self-rated health

Participants were asked to self-rate their health using the following question: Do you consider your health as being: Very good; Good; Medium/fair; Poor; Very poor? The participants who responded “good”, or “very good” were classified as “good SRH” (healthy), otherwise they were classified as “poor SRH” (unhealthy)^34–36^.

#### Disability

In this research, we did not aim to specifically explore the later stages of the disability process (i.e. the ADL-disability stage), so we chose to focus on the IADL-disability. Therefore, disability was measured through the Lawton’s scale, which assesses difficulties in performing the following instrumental activity of daily living (IADL)^13^: use the telephone, do the shopping, use transportation, handle medications, manage finances, prepare meals, do laundry and housekeeping (as recommended for this scale, the three last activities exclusively concerned women). The functional assessment conducted in the ecological context of the participant’s home enabled the neuropsychologist to adjust the conclusion (disabled or not) based on observation of the home (cleanliness and tidiness of the dwelling, handling of bills and administrative documents…), the participant’s general behavior, and the reactions of a close relative or a professional caregiver if present during the interview. As recommended by the authors, participants were considered IADL-disabled, if they were unable to perform at least one activity without human assistance, otherwise they were considered non-disabled^13^.

### Statistical analysis

All analyses were performed in the pooled sample. To estimate HEs, we used a maximum likelihood computer program using Interpolated Markov Chain (IMaCh, version 0.99r43)^37–39^. This program is based on a multinomial logistic regression model and was designed to analyze four transitions between three possible states (Healthy status → Unhealthy; Unhealthy → Healthy; Healthy → Death; and Unhealthy → Death). The minimum data required for the program are those from participants interviewed at baseline who had at least one available follow-up data (health indicator or death). Noted that, this program has the advantage of accounting for the varying time between waves or missing waves, using interpolation or extrapolation, therefore no information is rounded or lost^37,38^.

The multi-state model ran on three states: healthy, unhealthy, and death, to compute TLE, HLE, and UHLE by age and sex for SRH and TLE, DFLE and DLE for disability. To check whether the trends from the pooled sample were consistent between cohorts, a sensitivity analysis was conducted by replicating the analyses in each cohort.

Noted that, to test whether our two variables of interest (SRH and disability) were independent (not associated), we performed the Chi-square test. In addition, to measure how strongly these categorical variables were associated, we calculated Cramér’s V to measure the effect size. These analyses were also performed with regard to sex. Furthermore, an exploratory analysis was conducted to examine the extent to which the estimated HEs were correlated beyond the relationship between the observed measures of SRH and disability. We used R software (version 4.1.2) for the descriptive analysis.

## Results

The study sample included 4468 participants, of whom 2710 (61%) had more than four repeated assessments. Details of repeated assessments per study participant are shown in Fig. S1.

Of the total of 4468 participants, 2520 (56.4%) were women and 1948 (43.6%) were men. Participants’ median age at baseline was 77.00 years [interquartile range: 72.00–81.00]. The maximum time of follow-up was 21 years, and the total number of person-years was 35,378. The median time of participation was 10 years [interquartile range: 6–16] in 3C, 6 years [interquartile range: 3–10] in AMI, and 5 years [interquartile range : 2–10] in the PAQUID cohorts. A total of 2932 (65.62%) participants died during the follow-up (Table S1). Baseline characteristics of participants according to their status at the end of the study are shown in Table S2. Participants who dropped out of the study had profiles closer to (but not as good as) those who were still alive at the end of the study. Only those who died during follow-up had worse conditions at inclusion.

At baseline, 3249 (73.5%) participants were IADL-disability free, and 2285 participants rated their health as good (51.7%). The measures of SRH and disability were significantly and moderately related (*p*-value < 0.001 and Cramér’s V = 0.234). Across the whole pooled sample, 67.80% of disabled participants rated their health as poor compared with 41.20% of the non-disabled ones. Table 1 provides the distribution of SRH by Disability status in the pooled sample and age subgroups, at baseline (Table 1).

**Table 1:** Distribution of self-rated health by disability status in pooled sample and age subgroups, at baseline.

Disability measure	Self-rated Health measure			*p*-value of the Chi-squared test	Cramer’s V
	Good SRH	Poor SRH	Total		
Pooled sample (N = 4468)				**< 0.001**	0.234
Disability-free	1909 (58.8)	1340 (41.2)	3249		
Disability	376 (32.2)	792 (67.8)	1168		
Total	2285	2132	4417^+^		
Age ≤ 75 (N = 1719)				**< 0.001**	0.183
Disability-free	929 (60.0)	620 (40.0)	1549		
Disability	43 (28.3)	109 (71.7)	152		
Total	972	729	1701*		
Age [75–85] (N = 2099)				**< 0.001**	0.245
Disability-free	857 (57.4)	635 (42.6)	1492		
Disability	177 (30.2)	409 (69.8)	586		
Total	1034	1044	2078^$^		
Age ≥ 85 (N = 650)				**< 0.001**	0.216
Disability-free	123 (59.1)	85 (40.9)	208		
Disability	156 (36.3)	274 (63.7)	430		
Total	279	359	638^#^		

^+^51 Missing data; *18 Missing data; ^$^21 Missing data; ^#^12 Missing data.

Significant values are in bold.

In the pooled sample, the proportion of poor SRH and disability at baseline was significantly higher in women than in men, with a small effect size (weak association). The results were relatively similar for the difference in the proportion of poor SRH between the two sexes in the oldest age group (48.3% in men versus 60.8% in women). In contrast, in the oldest age group, the difference in the proportion of disability between the two sexes was more pronounced (51.9% in men vs. 76.6% in women), but with a moderate effect size (moderate association). (Table 2).

**Table 2:** Baseline distribution of self-rated health and disability by sex, in pooled sample and in the oldest participants.

Variables	Sex		Total	*p*-value of the Chi-squared test	Cramer’s V
	Men	Women			
*Pooled sample*					
Poor Self-rated Health				**< 0.001**	0.112
Yes	810 (41.9)	1330 (53.2)	2140		
No	1125 (58.1)	1172 (46.8)	2297		
Total	1935	2502	4437^#^		
Disability				**< 0.001**	0.107
Yes	412 (21.3)	771 (30.8)	1183		
No	1526 (78.7)	1734 (69.2)	3260		
Total	1938	2505	4443^+^		
*Age* ≥ *85 (N* = *650)*					
Poor Self-rated Health				**0.002**	0.121
Yes	112 (48.3)	248 (60.8)	360		
No	120 (51.7)	160 (39.2)	280		
Total	232	408	640^##^		
Disability				**< 0.001**	0.254
Yes	122 (51.9)	315 (76.6)	437		
No	113 (48.1)	96 (23.4)	209		
Total	235	411	646^++^		

^#^31 missing data; ^+^25 missing data; ^##^10 missing data; ^++^4 missing data.

Significant values are in bold.

### Total life expectancy by sex

**Figure 2:**
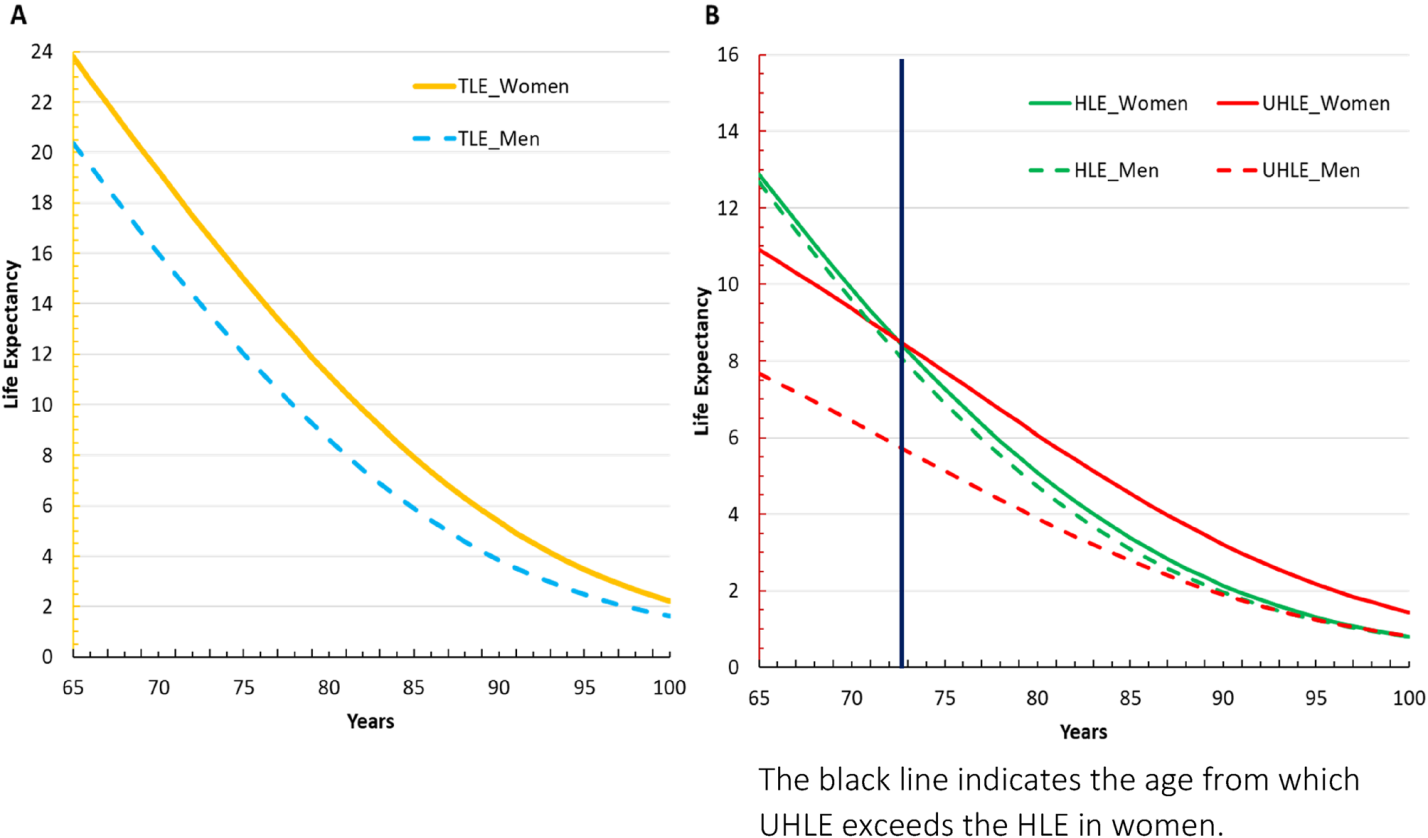
Life expectancy in good and poor self-rated health by sex, in pooled sample. (**A**) Total life expectancy by sex. (**B**) LE to be spent in good and poor self-rated health by sex.

Figure 2 shows the TLE and the number of years to be spent in good and poor SRH (HLE and UHLE) by sex.

As expected, women lived longer than men; and the sex gap in TLE decreases with age in absolute terms and increases in relative terms. Women had a + 3.43 years longer TLE than men at age 65 (in proportion, women can expect to live + 14% of TLE), and + 2.04 years at age 85 (+ 26% of TLE) (Fig. 2A).

Regarding, HLE and UHLE, men and women could expect to live almost the same duration in good SRH, whatever the age. However, compared to women, men could still expect to live longer in good than in poor SRH whatever the age. Indeed, in men, HLE remains higher than UHLE at all ages; while in women, UHLE exceeds HLE from age 73 onwards, as shown by the black vertical line in Fig. 2B.

### Comparisons of subjective health expectancy estimates (SHR) with those based on disability (IADL) in men and women

**Figure 3:**
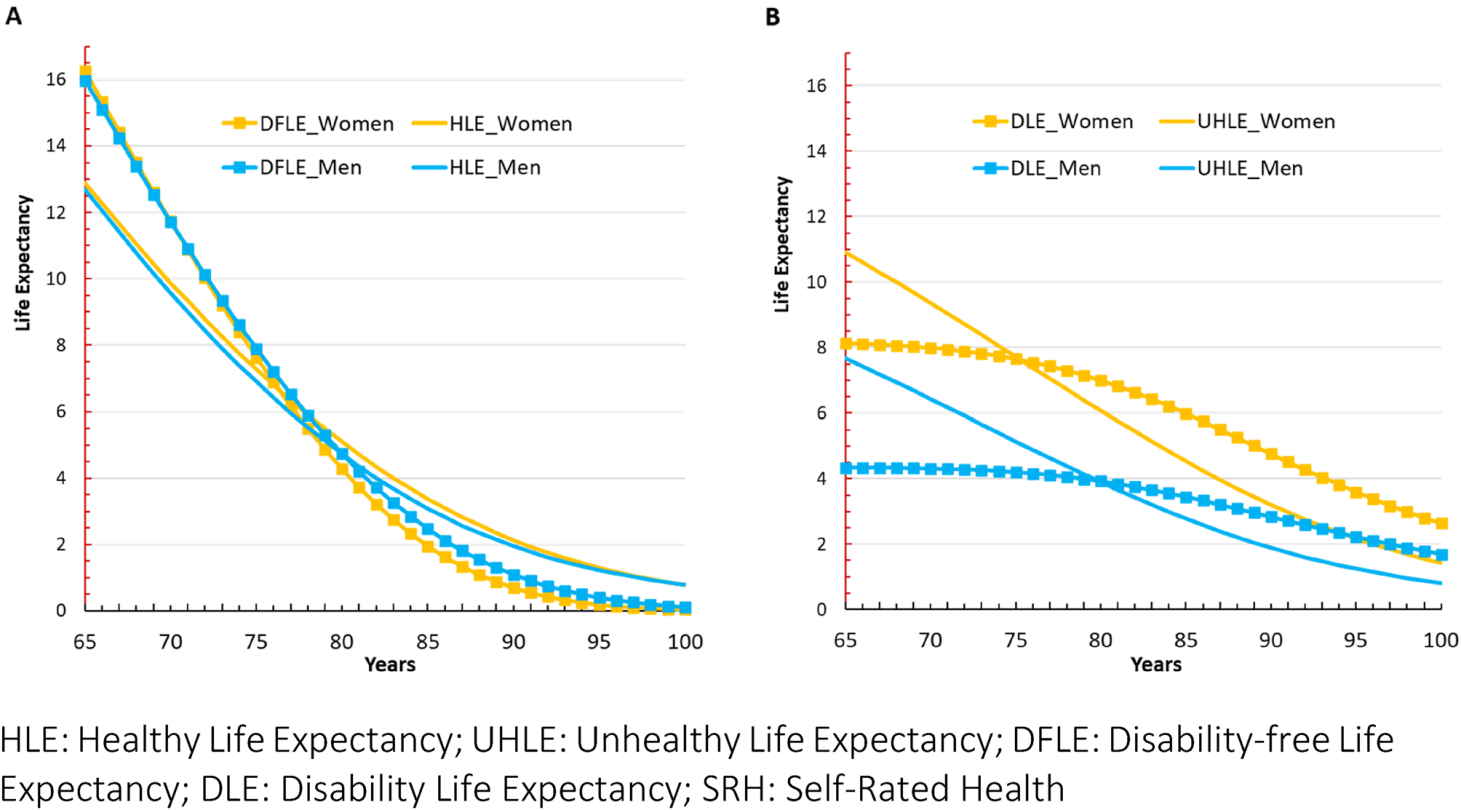
Age-evolution of the life expectancy in good/poor SRH and without/with disability by sex, in pooled sample. (**A**) LE in good health by sex SRH virus disability. (**B**) LE in poor health by sex SRH versus disability.

Consistently with the previous results on HLE, DFLE in men and women were quite similar at all ages (Fig. 3A). Nevertheless, a sex-pattern was observed when comparing UHLE and DLE. Indeed, while UHLE exceeded DLE at younger ages (+ 2.86 in women and + 3.32 in men, at age 65), a reversal was observed with an earlier tipping age in women (76 vs. 80 in men) (Fig. 3B). From these tipping ages, the proportion of the remaining lifetime in good health decreased with age, especially for disability-free (Fig. 3A and Table 3).

**Table 3:** Repartition of life expectancy in good/poor SRH and without/with disability at ages 65, 75 and 85 for women and men, in pooled sample.

Ages (years)	Health expectancy based on SRH indicator			Health expectancy based on disability indicator		
	HLE	UHLE	% of UHLE	DFLE	DLE	% of DLE
*Women*						
At age 65	12.88	10.91	45.85	16.21	8.05	33.18
At age 75	7.27	7.72	51.53	7.55	7.55	50.00
At age 85	3.38	4.54	57.32	1.95	5.96	75.34
*Men*						
At age 65	12.69	7.67	37.67	16.01	4.35	21.36
At age 75	6.90	5.14	42.73	7.89	4.15	34.46
At age 85	3.09	2.79	47.45	2.45	3.44	58.40

*HLE* healthy life expectancy; *UHLE* unhealthy life expectancy; *DFLE* disability-free life expectancy; *DLE* disability life expectancy.

As presented in Table 3, in women, at age 65, UHLE was 10.91 years (45.85% of TLE) vs. 8.05 years for DLE (33.18% of TLE). At age 75, the estimates became similar (both proportions around 50%) before DLE exceeded UHLE at older ages. In men, the estimates of poor health were lower than that in women. At age 65, the UHLE in men was 7.67 years (37.67%) versus 4.35 years for DLE (21.36%). At age 75, in contrast to women, the UHLE remained higher than the DLE, before becoming lower from age 80 onwards (Table 3).

Regarding the exploratory analysis of correlations, we observed a strong positive linear relationship between UHLE and DLE (estimate ≥ 0.92 in both sexes), and between HLE and DFLE (estimate = 0.99 in both sexes). The results were similar for the relationship between UHLE and HLE (estimate = 0.99 in both sexes) and between DLE and DFLE (estimate ≥ 0.84 in both sexes).

In sensitivity analyses, examining each cohort separately (Fig. S2), we observed that in the AMI cohort, women had a longer DFLE than men at all ages. Except this difference, the other observations were in line with the main results.

## Discussion

Based on three southern French population-based cohorts on aging, the present study shows the existence of sex differences in the time to be spent in poor SRH across ages, whilst no differences were found regarding expected lifetime in good health. While the proportion of UHLE increased with age in both sexes, women had longer TLE and UHLE but similar HLE as men at all ages. When comparing the life expectancies calculated using the subjective health and the disability indicators (considered as a more objective indicator), the study reveals specific results by indicator and different trends by sex across ages. Indeed, the UHLE was longer than the DLE for young older people (with a larger gap in men) and shorter for older people (with a larger gap in women). As for good SRH, lifetime lived free of IADL-disability was similar in both sexes. It should be noted that, in both sexes, the HLE was lower than the DFLE at younger ages but became higher at older ages, with a larger and earlier gap in women. In addition, the proportion of DLE in TLE increased substantially with age (especially up to age 75 for women), whereas this dynamic was less important for the proportion of UHLE in TLE, which increased less rapidly and similarly for both sexes.

### Sex differences in the expected years to be spent in poor and good SRH

As mentioned in introduction, the well-documented sex health-survival paradox supports that men and women have different health profiles. Women are more likely to have higher levels of morbidities (e.g., hypertension, depressive symptoms), poorer socioeconomic conditions, and chronic conditions that are often non-fatal but disabling (e.g., dementia, arthritis, frailty)^4,5,25,40–42^. In contrast, men are more likely to suffer from fatal diseases (e.g., heart disease, stroke)^6,43^. Although we did not use multimorbidity as an indicator of health in this study, our results confirm this paradox by showing that compared to men, women can expect to live longer but with poorer subjective health and in disability.

Our findings are consistent with previous research which used SRH as health indicators to compute HLE and UHLE^30,34,44,45^. Despite differences in TLE between studies, all results showed that women could expect to live longer and in poorer SRH than men^30,34,44,45^. Like our study, other studies have also reported that, while the expected number of years spent in good SRH was relatively similar between men and women, the proportion of HLE in TLE remained higher in men than in women^30,45^. However, in contrast to our sex-specific trends in HLE and UHLE, Belon et al. found among Brazilians older adults that women would spend their additional TLE in good rather than in poor SRH (e.g., at age 65: in women, 17.1 HLE out of 19.6 years of TLE vs. 13.5 HLE out of 15.6 years of TLE in men). They also observed that women and men would spend similar time in poor SRH (difference =  + 0.4 years at age 65 and + 0.1 at age ≥ 80)^29^. Such findings differences with our study may reflect contrasted living conditions and culture (e.g., how people perceive aging? what does “being in good or poor health” in old age mean to them? what are the sex-specific social norms?). Indeed, as mentioned earlier, SRH measure includes/reflects also cultural specificities.

### Comparisons of life expectancy dynamics based on SRH and disability indicators

We found that for both sexes, even though the HEs were correlated and decreased with age, the estimates computed using the disability indicator were almost systematically different from those obtained using the SRH indicator. The findings reinforce the fact that, although moderately related (medium effect size), SRH and disability remain complementary health measures for understanding and addressing the biopsychosocial conditions of individuals as they age. The present study provides an original contribution to the literature on the HEs by exploring the sex-specific patterns in the age trends of life expectancy spent in good and poor health calculated using SRH and disability indicators.

Our study highlights important age differences in the trends of HE based on SRH and disability indicators. Indeed, we found a reverse pattern in the trends of the expected time to live in poor SRH and in disability, across ages. Regardless of sex, the expected lifetime in poor SRH was longer than that in disability at youngest ages and becomes shorter with increasing age. Some hypotheses could explain these findings. Firstly, in early old age (before age 70 or 75), individuals are more likely to report poor health conditions without necessarily needing help to perform the activities of daily living. For example, in our analysis sample, at baseline, of the 729 people aged ≤ 75 who reported poor SRH, only 109 (15%) had reported IADL difficulties. At older ages, we observed situations where more people have disability but did not necessarily consider their health as poor. We can assume that the ‘young old’ are probably more critical of their health than the ‘oldest old’, who consider it ‘normal’ to experience limitations and declines in old age and therefore report relatively good subjective health despite disability. Therefore, they would not consider themselves to be in such a poor health even if they have IADL disability, since they are almost like their counterparts or healthier than those who are worse off (“downward social comparisons”). This supports the fact that SRH also reflects the differences in the individual’s current conditions and past experiences given their age and compared to other people they may know, as proposed by Jylhä M.^21^. Secondly, the reversal trend observed with age indicates that the presence of disability with advancing age does not necessarily nourish the feeling of being in poor health. Indeed, on the one hand, it can be hypothesized that if these limitations do not prevent individuals from continuing the activities they value despite advanced age (e.g., receiving visits from family or friends, playing cards, cooking…), they may feel less unhealthy than at younger age. On the other hand, in the presence of limitations, older people may reduce their standards for good subjective health (recalibration response shift) and/or their priorities (reprioritization response shift)^27^. Therefore, from a public health perspective, SRH and disability measures in older adults are not interchangeable but appear to be complementary. In addition, depending on the research or health system question issue, it may be appropriate to use these measures in combination or independently. From the perspective of healthcare and long-term care planning, it may be advisable to use disability measures, whereas from the perspective of monitoring the consequences of social inequalities in health, it may be more relevant to understand the dynamics of poor subjective health measures. Identifying people who are not yet disabled but who perceive themselves to be in poor health could also help to address related needs to improve well-being, quality of life and reduce the risk of health deterioration.

The present study also highlights sex differences in the trends of DLE and UHLE, across ages. Indeed, DLE became higher than UHLE earlier in women than in men. This result can be explained by the fact that women seemed to have a higher standard of subjective health at a younger age and therefore had a higher proportion of UHLE (46% of TLE at age 65) than men (38% of TLE at age 65). With age, women tended to revise their standard of subjective health downward, reducing the proportion of their remaining life expectancy in poor subjective health, even though more than half of their total remaining life expectancy will be spent in disability from age 75 onwards. This change in the standard of subjective health appears to occur at an older age in men.

### Strengths and limitations

To our best knowledge, this is the first study that investigated sex-specific pattern in HLE based on subjective health and DFLE across older ages and to compare the two. We thus showed the extent to which the indicator used to assess health status can affect estimates of HEs. Therefore, this study provides original additional contribution in the HEs research in older adults. Secondly, we used a multi-state modelling analysis suitable for longitudinal data. Since longitudinal data, such as ours, cover a relatively long period, and that health behaviors and care environments can change, the probabilities of dying and/or being in good or poor health can also change. Therefore, it is important to emphasize that unlike prevalence-based methods, the IMaCh program used the current incidence of all possible transitions between states (including recovery) and the dynamics of survival rates over time to provide TLE and more realistic HEs^37–39^. Thirdly, we did not exclude from our analyses the participants who entered in an institution in later survey waves, as they are still being followed.

Despite these strengths, some methodological choices may have impacted our results and need to be discussed. Firstly, to define the poor SRH group we combined individuals who self-rated their health as fair, poor, and very poor. This classification may have influenced our estimates by underestimating the UHLE because participants who self-rated their health as fair could be considered as being in relatively good health. However, our classification is similar to that used in several studies^34–36^. Secondly, as the sample is not representative of the general French population and as a recent study has shown, the southern department to which Bordeaux belongs is one of those with the highest LE among the 100 French departments (25th /100 for women’s LE at age 60 and 22nd/100 for men)^46^, we cannot extrapolate our results to the French population as a whole. Thirdly, we observed some specific patterns in the AMI cohorts (retired farmers). Contrary to the other cohorts, DFLE was longer in women than in men that could be explained by different selection criteria according to sex. Indeed, only farmers insured in their own name could be included from the farmers’ health insurance reimbursement database (men were more likely to be in this situation). Women insured in their own name (potentially included in the sample) may have been different from women insured under their husbands’ contracts (not enrolled in the study). They may be more likely to be farm owners who are therefore not representative of all women in agriculture. Although this result differs from that of the two other cohorts it did not alter our conclusions. However, it should be noted that by pooling the three cohorts, we have sought to consider, to some extent, the different profiles that can be found in the general population (e.g., people living in rural, urban areas, being retired farmers). Moreover, as the design of these cohorts and the definition of the two health indicators were similar, pooling these cohorts also allowed us to have a reasonable sample size for stratification by sex, and increased the statistical power of our analyses. Fourthly, as there is a gap of several years between the real starting points of our three cohorts, the participants do not belong to the same birth generation, it is important to note that beyond age, perceptions of aging may have evolved from one generation to the next, leading to changes in the importance people attach to the different factors taken into account when self-assessing their health. Therefore, we cannot rule out a possible cohort effect in the change observed in the dynamics of the DLE and of the UHLE. However, the results regarding the age of change in the pooled sample and in the individual cohorts were consistent (the tipping point is between 75 and 77 years of age depending on cohort). Finally, our measure of IADL disability was less objective than that which can be obtained from a performance-based test. It is also important to stress that although the (Lawton validated) scale used, included the assessment of three additional IADLs items in women, this sex-specific assessment did not increase the probability of disability in women compared to men. Indeed, using the same scale, it has been shown that individuals enter into IADL disability through limitations in shopping and transportation, two IADL items that are common to both sexes^47^. In women, limitations in the three additional IADLs items occur later. In this study, as we were not interested in the number of IADL limitations presented by participants, the effect of this sex-specific assessment of IADL disability on our results was practically null. Our results showing that women had a higher DLE than men are consistent with the extensive literature on sex differences in DLE/DFLE^14,34,48,49^.

## Conclusion

This study highlights the existence of substantial differences in the dynamics of subjective and disability health expectancies across age and between men and women. When looking at different stages of the aging process, feeling in poor health status does not systematically mean that the individual is IADL-disabled (especially among the young old), and having an IADL-disability does not systematically lead to feeling in poor health (especially in the older old). However, these two measures are related, and our findings reinforce the relevance of using subjective indicators in addition to objective health indicators in research on HEs to monitor trends and inequalities in healthy aging (e.g., by age, sex) to highlight the health needs with advancing ages. Indeed, as emphasized by the World Health Organization in the concept of healthy aging, a person-centered approach based on the subjectivity of health is also central to a comprehensive vision of aging.

### Supplementary Information

Supplementary Information.

## Data Availability

The datasets generated and/or analyzed during the current study are not publicly available as there are still ongoing scientific valorization but are available from the corresponding author on reasonable request.
