# Supplementary material for: Differences between subjective and disability health expectancies across ages in older adults
Source: Sci Rep. 2024 Jun 26;14:14731. doi: 10.1038/s41598-024-65416-3 (PMC11208525; doi:10.1038/s41598-024-65416-3)
Supplement: Supplementary file 1 — Supplementary Information. [file 41598_2024_65416_MOESM1_ESM.docx]

**Figure S1:** Details of repeated assessments per study participants.

Note: Except for the first measurement, the number of participants reported for the other measurement includes incident deaths occurring after the last data collection wave, as they contribute to the calculation of total life expectancy.

**Table S1:** Repartition of the number of person-years and participation time in the pooled sample and per cohort.

| **Cohorts** | **Total sample size analyzed (% of women)** | **Median age [Q1 – Q3]; in years** | **Statistics of participation time** | | | **Number of deaths recorded** |
| --- | --- | --- | --- | --- | --- | --- |
|  |  |  | **Total person-years**  **(for women)** | **(Min-max); in years** | **Median**  **[Q1 – Q3]; in years** |  |
| AMI | 978 (37%) | 76 [71 – 81] | 5,959 (2,413) | (0 – 12) | 6 [3 - 10] | 378 |
| 3C | 2,101 (61%) | 74 [71 – 78] | 20,624 (13,503) | (0 – 18) | 10 [6 – 16] | 1,241 |
| PAQUID | 1,389 (62%) | 81 [78 – 86] | 8,795 (5,852) | (0 – 21) | 5 [2 – 10] | 1,313 |
| Pooled sample | 4,468 (56%) | 77 [72 – 81] | 35,378 (21,768) | (0 – 21) | 7 [3 – 12] | 2,932 |

**Table S2:** Baseline characteristics of participants according to their status at the end of the study.

| **Baseline characteristics** | **Status at end of the follow-up** | | | **p-value** | **Effect size coefficient** |
| --- | --- | --- | --- | --- | --- |
|  | **Alive (n=382)** | **dropout (n=1154)** | **Died (n=2932)** |  |  |
| age, median [IQR], in years | 71 [69 - 75] | 73 [69 - 77] | 79 [76 - 84] | <0.001^$^ | 0.23^+^ |
| Sex Female, n(%) | 256 (67.0) | 651 (56.4) | 1613 (55.0) | <0.001 | 0.067^#^ |
| Poor Self-rated Health, n(%) | 142 (37.1) | 479 (41.5) | 1519 (51.8) | <0.001 | 0.11^#^ |
| Disability, n(%) | 16 (4.2) | 125 (10.8) | 1042 (35.5) | <0.001 | 0.286^#^ |
| Participation time, Median  [Q1 – Q3]; in years | 17 [16 – 17] | 9 [6 – 11] | 6 [2 – 10] | <0.001^$^ | 0.29^+^ |

^+^Eta squared; ^#^Cramer's V; ^$^Kruskal-Wallis rank sum test.


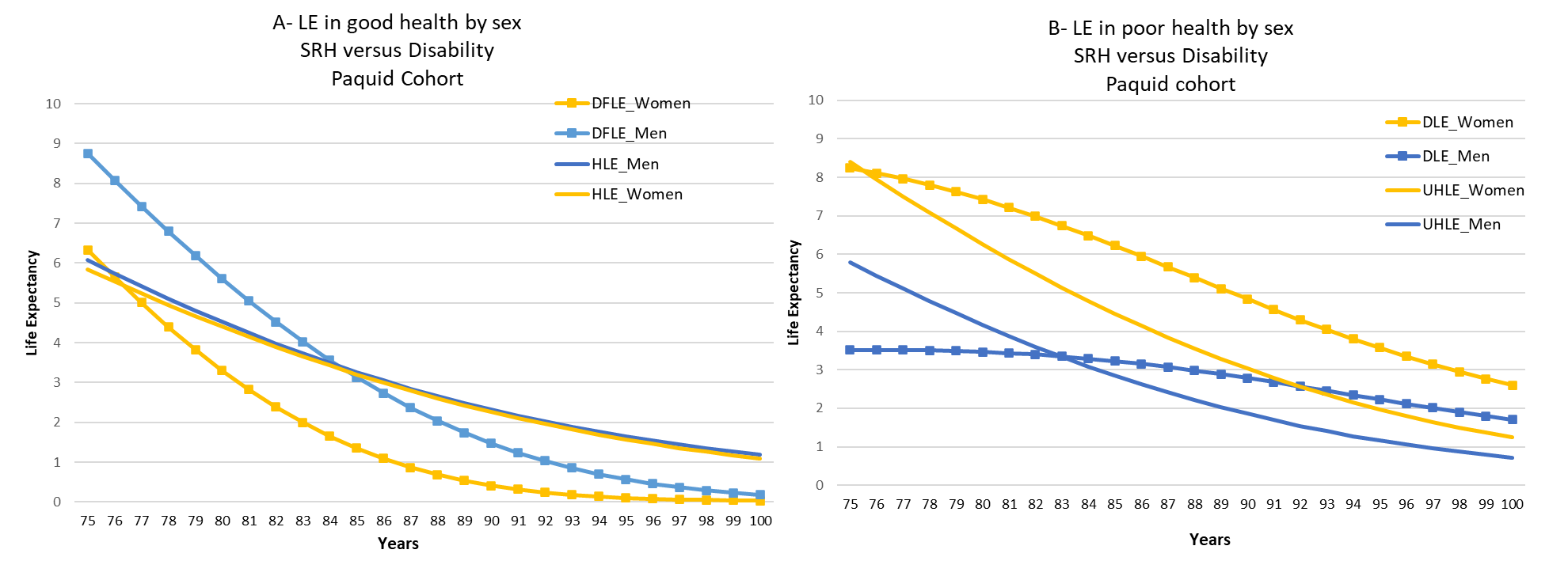

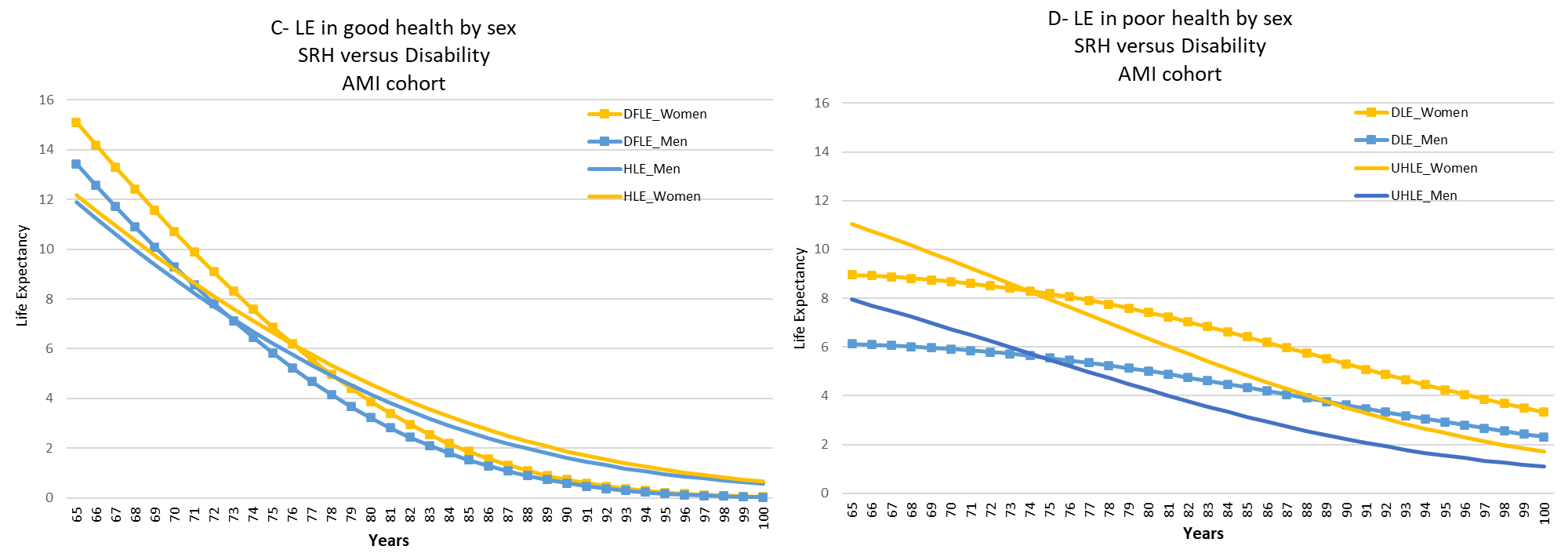

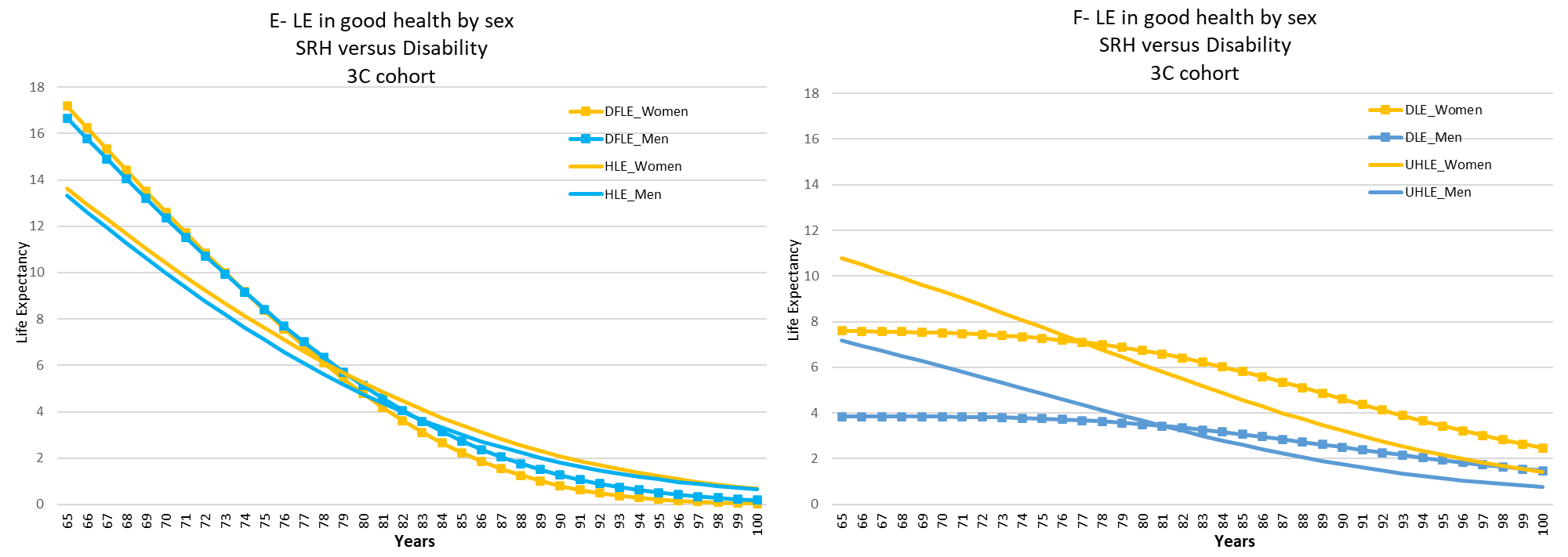


**Figure S2**: Age-evolution of the life expectancy in good/poor SRH and without/with disability by sex, in each study cohort.
